# Recent advances in understanding DNA replication: cell type–specific adaptation of the DNA replication program

**DOI:** 10.12688/f1000research.15408.1

**Published:** 2018-08-29

**Authors:** Antoine Aze, Domenico Maiorano

**Affiliations:** 1 Institute of Human Genetics, UMR9002, CNRS-University of Montpellier, Montpellier, 34396 Cedex 5, France

**Keywords:** DNA synthesis, nucleus, chromatin, epigenetics, development, cell cycle, differentiation

## Abstract

DNA replication is an essential process occurring prior to cell division. Cell division coupled to proliferation ensures the growth and renewal of a large variety of specialized cell types generated during embryonic development. Changes in the DNA replication program occur during development. Embryonic undifferentiated cells show a high replication rate and fast proliferation, whereas more differentiated cells are characterized by reduced DNA synthesis and a low proliferation rate. Hence, the DNA replication program must adapt to the specific features of cells committed to different fates. Recent findings on DNA synthesis regulation in different cell types open new perspectives for developing efficient and more adapted therapies to treat various diseases such as genetic diseases and cancer. This review will put the emphasis on recent progress made in this field.

## Introduction

DNA synthesis occurs during the S phase of the cell cycle and is ensured by the replisome, a molecular machine made of a large number of proteins acting in a coordinated manner to synthesize DNA at many genomic locations, the replication origins ^1^. Replication origin activation in space and time (or replication program) is set by a sequence of events, starting already at the end of mitosis, lasting through G _1_ phase, and ending in S phase when DNA replication is activated. These coordinated events ensure that the full genome will be replicated before mitosis since a faithful DNA synthesis is a prerequisite for genome integrity maintenance ^2^. DNA replication initiates from thousands of replication origins scattered along the chromosomes. Origins acquire the competence to replicate in a step called “licensing” that involves formation of a pre-replication complex (pre-RC), including loading of the replicative helicase MCM2-7 (recently reviewed 3). Then once the transition from pre-RC to a pre-initiation complex (pre-IC) is induced by S-phase kinases, DNA replication is activated and DNA is unwound to provide the template for the replicative DNA polymerases ^2, 4^.

The last 30 years of deep investigations in the DNA replication field have allowed the general mechanisms involved in eukaryotic DNA synthesis to be defined. Thanks to recent methodological improvements, such as genome-wide analysis ^5, 6^, *in vitro* reconstitution assays ^7– 9^, proteomics ^10– 12^, and structural biology ^13, 14^, our vision of the molecular pathways that govern DNA synthesis is becoming sharper.

Although the factors that drive DNA replication initiation are well conserved throughout the eukaryotic kingdom, cell type–specific differences in the regulation of this process within eukaryotes have been recently unraveled. From these studies, it appears that the regulation of DNA replication is influenced by the fate of a given cell. These findings shed new light on how the DNA replication program and the proliferation rate are being modulated during cell fate commitment, when cell type specialization is determined. New discoveries in this topic are undoubtedly essential to expand our understanding about the genesis of diseases and their progression. In this review, we will briefly describe recent achievements aiming to understand how the replication factors involved in the licensing, activation, and elongation steps of DNA synthesis adapt dynamically with cell fate determination to maintain genome integrity and homeostasis.

## Coordination between replication origin licensing and G _1_ length is critical for cell destiny

**Figure 1.**
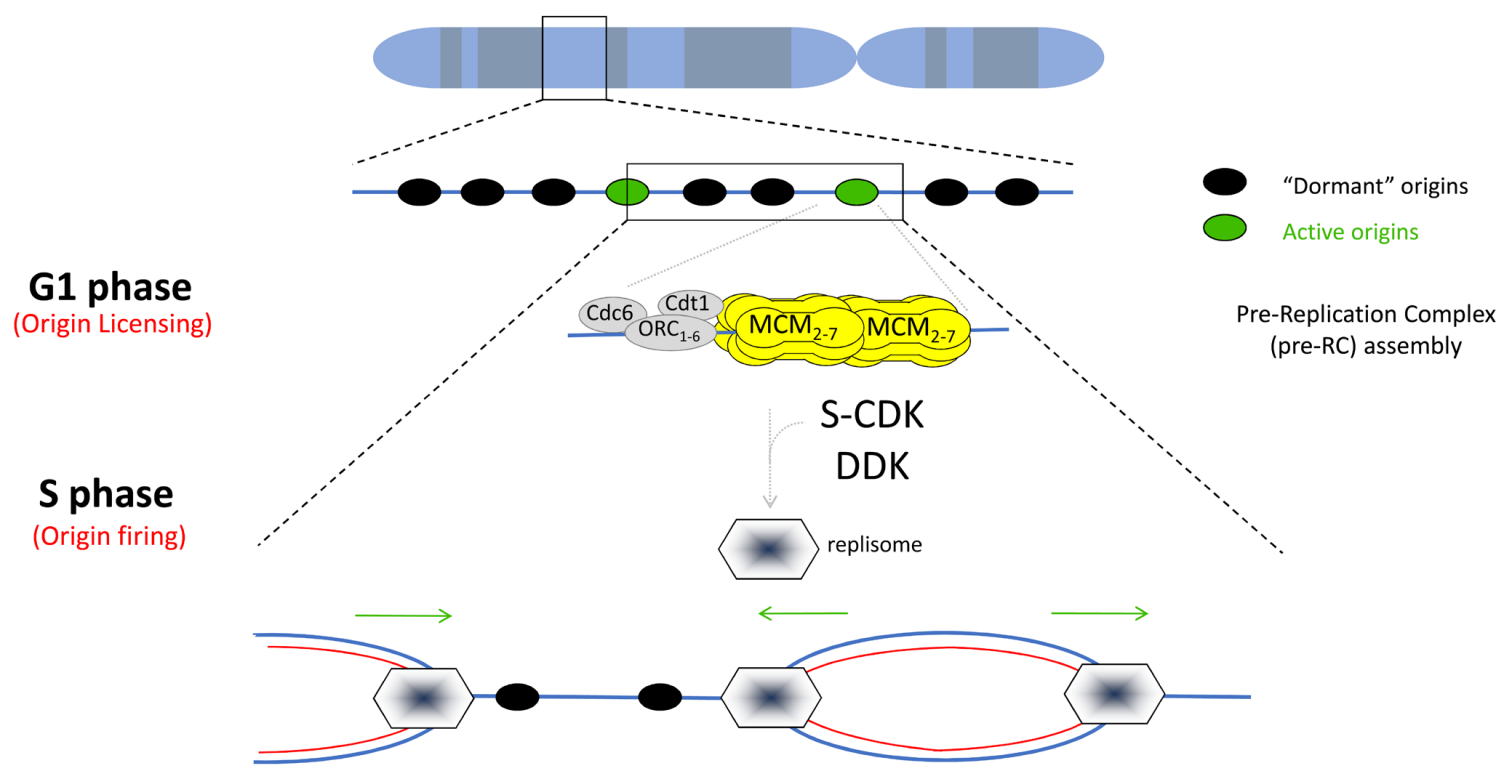
Replication of eukaryotic chromosomes. Replication origins are scattered along the genome to ensure that each chromosome is entirely replicated in S phase. Pre-replication complex (Pre-RC) assembly and origin activation are tightly regulated in a sequential manner to ensure that replication occurs only once per cell cycle. Hence, throughout the G _1_ phase, origins are licensed by the sequential loading of the pre-RC components: Origin Recognition Complex (ORC), CDC6, CDT1, and (at the final stage) MCM2-7. Once a double MCM2-7 hexamer is stably recruited onto chromatin, origins are licensed. Many replication origins are licensed but few are activated, allowing a backup of origins to be used when DNA replication is perturbed. Origin firing occurs under the combined activities of S-phase Cyclin-Dependent Kinase (CDK) and Dbf4-Dependent Kinase (DDK), allowing the recruitment of additional factors involved in DNA replication initiation and elongation. MCM2-7, together with CDC45 and GINS, composes the DNA helicase that unwinds DNA in a bidirectional manner. The entire replication machinery made of accessory factors required for replication fork stability and the synthesis of DNA is called the replisome.

*In vitro* reconstitution assays in yeast have provided tremendous detailed information on how origins are licensed by dissecting the sequential biochemical steps involved ^3, 7– 9, 14, 15^. Detailed reviews covering the main features that characterize metazoan origins have been recently published ^3, 5, 16^. Briefly, in eukaryotes, replication origins are licensed through a chronological order that requires the binding of Origin Recognition Complex (ORC), Cdc6, and Cdt1 proteins onto chromatin ^17, 18^. Two hexamers of the MCM2-7 helicase complex then are loaded in an inactive state prior to S phase ( Figure 1). In yeast and mouse cells, Cdt1 and MCM2-7 form a complex before being recruited onto replication origins by ORC and Cdc6 ^19– 21^. The MCM2-7 double hexamer must be activated by S-phase kinases to be able to unwind DNA and initiate DNA replication. However, MCM2-7 complexes are recruited in excess so that not all MCM2-7 complexes are activated during a cell cycle ( Figure 1). The choice of the origins to be activated (around 30,000 in mammalian cells) is variable from cell to cell and ensures flexibility in origin usage during the DNA replication program to adapt with cell fate commitment, cell environment, and replicative stress ^22, 23^. Origin mapping strategies coupled with high-throughput sequencing revealed that the chromatin context influences origin selection through genetic and epigenetic features located in close proximity to the origins.

It is now clear that most replication origins being constitutively activated across multiple metazoan cell types (hereafter named “shared origins”) are associated with unmethylated CpG islands, G-rich elements, transcriptional start sites, and histone modifications related to open chromatin marks (H3K4 ^me3^, H3K9 ^Ac^, and H3K27 ^Ac^) ^24– 29^. Notably, this population of shared origins tends to initiate early during S phase, whereas cell type–specific origins initiate during late S-phase and are linked with compacted chromatin marks ^28^. These observations underline the relationship between chromatin modifications with the cellular context in origin selection.

Two independent studies describing reconstitution of DNA replication initiation events *in vitro* from chromatinized templates in yeast provided biochemical evidence that the regulatory functions of chromatin structure influence origin selection. These studies confirmed that nucleosome-free regions contribute to defining ORC binding and thus origin function ^30, 31^, as previously suggested from genome-wide studies mapping ORC binding sites and nucleosome occupancy in various organisms ^32– 36^. Moreover, in mouse embryonic stem (ES) cells, depletion of the histone H1 perturbs the landscape of replication origin activation ^37^. Because chromatin environment changes during cell fate commitment and in different cell types, it is generally assumed that the DNA replication program is coordinated with transcription to avoid transcription–replication conflicts and thus preserve genomic integrity. This seems particularly crucial during the onset of developmental programs and cell lineage specification. In *Caenorhabditis elegans*, origins from rapidly replicating pluripotent embryos coincide with open chromatin regions of highly transcribed genes, whereas establishment of the new transcriptional program, occurring at later embryonic stages when cell differentiation begins, correlates with the reorganization of replication initiation sites ^29, 38^.

Permissive chromatin features for rapid activation of replication origins could influence the cell cycle length. ES cells have a shorter G _1_ compared with their differentiated counterparts, which impacts on the licensing step. ES cells recruit much more MCM2-7 to the chromatin than tissue-specific stem cells or progenitor cells ^39^. This excess of origins that remain dormant during S phase appears to be important to maintain pluripotency ^40, 41^ and could also protect the genome against DNA replication stress occurring in ES cells ^39, 42^.

Besides changes in the kinetics by which MCM2-7 is loaded onto chromatin, licensing control adapts to G _1_ length, suggesting that pre-RC binding is developmentally regulated. Indeed, quantitative single-cell analysis performed in human cells by Matson and colleagues demonstrated that origins are licensed faster in pluripotent cells compared with their isogenic differentiated counterparts and that loading of the MCM2-7 helicase slows down as G _1_ duration is extended ^43^. The authors revealed that high expression of the licensing factor Cdt1 was important for fast MCM2-7 loading rates, thus enabling ES cells to rapidly license origins prior to the G _1_-to-S phase transition. Similar to the need for additional dormant origins, fast licensing kinetics seems essential to ensure pluripotency in ES cells and induced pluripotent stem cells. This observation was corroborated by Carroll and colleagues in intestinal stem cells from adult tissues ^44^. The authors demonstrated that licensing is interconnected with the proliferative commitment of stem cells. They found that Lgr5 ^+^ intestinal stem cells, which contribute primarily to the renewal of the intestinal epithelium, reside mostly in a G _1_, unlicensed state, although they express MCM2 and other proliferative markers to a level equivalent to the licensed population. Interestingly, this unlicensed state correlates with an elongated cell cycle that could be considered as a backup mechanism to sustain proliferative fate decisions and tissue maintenance. Nonetheless, the link between chromatin structures and adaptation to G _1_ length in such a context remains to be determined.

Uncoupling of G _1_ length with licensing kinetics has been observed during oncogenic transformation. Overexpression of oncogenes, such as cyclin E and c-MYC, shortens G _1_ and forces cells to engage S phase earlier with incomplete licensing. Consequently, the replication program is perturbed, leading to accumulation of replication stress ^45, 46^. Origin usage following oncogene activation was recently investigated genome-wide. The authors found that in these conditions new initiation zones, which were normally suppressed by transcription in G _1_, appear in intragenic regions ^47^. These results also confirm an observation in *drosophila* showing that active transcription modulates MCM2-7 distribution ^48^. Nonetheless, how RNA polymerases inactivate and redistribute MCM2-7 remains to be determined. It has been reported that DNA translocases and RNA polymerases in yeast are able to push MCM2-7 along DNA, but similar processes have not yet been described in metazoans ^8, 49^. An emerging picture from these studies is that cell cycle length, local chromatin structure, and active transcription can modulate the extent of origin licensing.

## Activation of DNA replication in different cellular contexts: control in space and time matters

**Figure 2.**
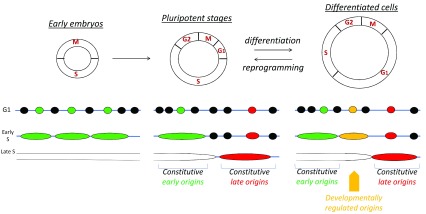
Replication program and cell features. Changes in the DNA replication program are dependent on cell features. In those rapidly dividing embryos, which are transcriptionally inactive, many replication origins fire to ensure fast S-phase. Selection of replication origins at this stage is believed to be random. Despite improvements in replication origin mapping, this idea has not yet been challenged. The timing of replication during early development is not clearly defined, as S-phase length is very short. During the pluripotency stage, appearance of gap phases and the onset of transcription confer a certain nuclear organization that sets the genome for a specific program of replication. Constitutive origins that replicate early are associated with strong origin density and efficiency, a high GC content, strong gene density, and nucleosome-free regions. On the other side, late constitutive origins are poor in origins and genes, GC content is low, and chromatin accessibility is restricted. Rif1 is a major factor shown to modulate origin activity for late replicating domains. The replication program changes dynamically during differentiation or cell lineage development, in coordination with changes in transcriptional activity and chromatin organization. Whereas constitutive origins are activated at the same location and with the same timing, some origins will be regulated following acquisitions of new cellular features. Remarkably, tumor development leads to formation of a heterogeneous population of cells, including cells with stem-like properties, progenitors, and differentiated tumor cells. These cells have the ability to perpetuate their lineage, to give rise to differentiated cells, and to undergo rapid growth. These molecular features, recapitulated during early embryonic development, illustrate similarities between cancer stem cells and embryogenesis. Understanding the DNA replication program regulation in a cell type–specific manner will allow investigation of the processes behind tumorigenesis from a different perspective.

During S phase, origins are activated by the combined activities of cyclin-dependent kinase (CDK) and Dbf4-dependent kinase (DDK) kinases ( Figure 1) targeting several substrates allowing the recruitment of CDC45 and GINS complex to MCM2-7, thereby forming the CMG complex, the functional replicative helicase ^50^. Chromatin environment contributes to licensing as described previously through MCM2-7 loading but also to origin selection through MCM2-7 activation ^51, 52^. The dynamics of origin activation during S phase follow a spatio-temporal order known as replication timing ( Figure 2). Replication timing in eukaryotes controls activation of replication of large chromosomal domains and is mediated by genetic determinants, local histone modifications, and global chromatin organization ^53^. Thus, DNA sequence features that mediate licensing in vertebrates can also affect the time when origins will be activated. Origin G-rich repeated elements (OGREs) are prone to form G-quadruplexes (G4s). Their presence in the genome is associated with origin activity ^25, 54^. Nucleosome organization at the proximity of origins was reported to modulate both origin licensing (as described above) and MCM2-7 helicase-dependent activation steps of initiation ^55^ in yeast and more recently in mammals ^37^. Different histone modifications and certain histone modifiers have been assigned in origin selection and the dynamics of their activation across S phase ^29, 38, 52, 56– 60^.

Several histone-modifying enzymes such as the methyltransferase PR-Set7 and the acetyltransferase HBO1 in complex with BRPF3 have been reported to have a role in activation of particular replication origins ^52, 61^. More recently, a great deal of investigation has focused on the contribution of chromatin regulators in controlling the timing of activation through chromatin organization. The telomere-associated protein Rif1 interferes with DDK-dependent phosphorylation of MCM2-7 and modulates nuclear architecture by anchoring heterochromatin ^62, 63^. Rif1 might organize replication-timing domains through its association with G4s to repress pre-RC activation until late S-phase ^63– 65^. The ORC-associated protein ORCA/LRWD1 establishes a repressive chromatin environment at a subset of origins, thus priming them for late replication ^66^. The replication-initiation determinant protein RepID initiates replication in a sequence-specific manner by interacting with a subgroup of origins ^67^.

Modulation of origin activation in a cell type–specific manner depends upon global changes in chromosomal architecture and mainly occurs within large chromosome domains known as Topologically Associating Domains (TADs) ^68– 71^. These constitute structurally distinct chromatin domains covering several megabases where DNA interactions are favored. TADs show a strong correlation with replication timing domains in which several replication units are concomitantly activated. Nuclear reorganization that accompanies differentiation and the establishment of developmental programs result in changes in replication timing of the human genome ^70, 72, 73^; notably, alterations in replication timing have been detected in several diseases ^74, 75^.

Therefore, flexibility in origin activation seems critical to accommodate the dynamic changes in the transcriptional program and genome repositioning ( Figure 2). Indeed, cell type–specific replication origins correlate with regions corresponding to differentiation-specific and tissue-specific gene expression programs ^76, 77^. Origins that are activated in a cell type–dependent manner appear to replicate late in S phase ^28^. Histones and chromatin modifications described above impose a crosstalk between replication and transcription and are interconnected during differentiation and development ^29, 38^ although this relationship remains enigmatic.

Origin activation dynamics that determine replication timing therefore might play a role in establishing local and global chromatin structure to facilitate the cellular response to the differentiation process. Remarkably, establishment of TADs during early development in mammals requires DNA replication but not transcription ^78^. Moreover, perturbations in DNA replication during development can cause epigenetic changes (alleviation of repressive marks) potentially inherited by the next generations ^79^.

Chromatin organization immediately following fertilization in vertebrates is particularly critical for DNA replication initiation and this occurs in a transcription-independent manner following fertilization ^80^.

The capacity to selectively modulate origin usage in a cell type–specific manner suggests that the proteins involved in origin activation might play specific functions. Indeed, replication initiation proteins, and their availability during S phase, can be involved in dictating specificity in origin activation ^81– 85^. The Sld3 vertebrate homolog Treslin/TICRR, a CDK target that acts as a binding site for TopBP1 and Mdm2 binding protein MTBP (both proteins being required for GINS-CDC45 recruitment), was proposed to link chromatin acetylation to DNA replication initiation efficiency and timing in different cancer cell lines ^58, 86^. This observation shows that Rif1 is not the only known site-specific regulator of DNA replication initiation and timing. Likewise, a function in origin efficiency has been assigned to the Treslin partner MTBP, probably through its ability to localize Treslin near G4 structures ^87^. Finally, Rif1 accessibility to chromatin was recently implicated in determining the onset of late replication during embryonic development ^88^.

Overall, these studies suggest a functional interaction between components of the replication machinery with chromatin modifiers leading to reorganization of the genome architecture during development. In turn, reorganization of the chromatin architecture defines cell type–specific transcriptional programs that feedback on the availability of replication proteins but equally on the replication timing by shaping chromatin architecture in specific domains such as TADs. Hence, genetic and epigenetic features set during developmental transitions may function as selective ways to repress or activate licensing or firing (or both) of a subset of origins, potentially within replication timing domains. Precise origin mapping at the single-molecule level in a single cell (using nanopore sequencing, for instance) completed by *in vitro* reconstitution assays using human proteins will undoubtedly bring to light new exciting concepts in this field.

## Adaptation of the replisome to fast proliferation

A large variety of cell types critical for tissue function are formed during early embryonic development, when cell commitment first takes place. The proliferative state decision is variable for each cell type and may influence cell cycle duration. Consequently, steps required for proliferation, such as genome duplication, must be tightly regulated with cell cycle progression to maintain homeostasis. For instance, embryonic cell division in metazoans exhibits dramatic lengthening of the cell cycle at the onset of gastrulation, a stage required for cell type specialization and embryo patterning ^89, 90^. Lengthening of the total cell cycle time is achieved mostly by extension of the G _1_ phase and moderately the S phase ^91^.

A variation in replisome composition following DNA replication perturbations or between different cell types was unraveled thanks to recent advances in quantitative proteomics at the replication forks ^11, 92, 93^. Analysis of the replication machinery at the forks by isolation of proteins on nascent DNA (iPOND) allows comparison of protein abundance at replication forks in different contexts. It was shown that replisomes of pluripotent stem cells contain a particular protein network that accommodates a high proliferative capacity with short cell cycle phases and reduced endogenous DNA replication stress ^94^. Factors involved in DNA repair, such as mismatch repair, but equally pluripotency and epigenetic inheritance factors, such as the NuRD-HDAC complex, were found to be enriched at replication forks. Interestingly, independent studies confirmed the requirement of a NuRD complex for DNA replication during early embryonic development ^95^. ES cells seem to require additional factors at the replication forks to cope with DNA replication perturbations. Thus, Filia-Floped was identified by iPOND as a new protein complex involved in resolution of stalled forks during a normal S-phase in mouse ES cells compared with their differentiated counterparts ^96^. It is likely that strategies set in stem cells to ensure fast DNA replication may also be exploited in other biological contexts requiring fast proliferation, including very early embryogenesis and tumorigenesis. For example, the Rad18 E3 ubiquitin ligase, a master regulator of translesion DNA synthesis, is an abundant component of the DNA replication machinery during *Xenopus* early development and confers to the replisome the ability to hijack DNA lesions and suppress the DNA damage response, ensuring fast cell cycle progression. Remarkably, Rad18 was found to be highly expressed in glioblastoma cancer stem cells ^97^.

Overall, these recent observations converge toward the idea that replisome composition can be selected “ *a la carte*” regarding the biological features set by specific cell types. Consistent with this idea, replication of specific genome locations like telomeres, common fragile sites, or centromeres has been shown to involve particular DNA replication partners at the vicinity of the DNA replication fork to ensure stability of those genomic regions ^98– 100^.

## Conclusions

A defective DNA replication program is the source of several pathologies during development (Meier-Gorlin syndrome) and during the adult life (carcinogenesis) ^45, 101^. Metazoan origins lack consensus sequences and are associated with different structural features that confer cell type–specific replication. Additional mechanisms operate to fine-tune pre-RC assembly, origin activity, and replisome composition. This allows cells to coordinate a fast replication program with their fate, contributing to genome integrity maintenance despite DNA replication perturbations often observed during tumorigenesis and development. Identification of various factors involved in origin selection and their activity in a cell type–specific manner is a great source of interest. Such factors may often be deregulated during cancer development; thus, their targeting might constitute effective anti-cancer therapies.

Nevertheless, these discoveries are only the tip of the iceberg as limited information is currently available regarding their regulation. Biochemistry, electron cryomicroscopy analysis, *in vitro* reconstitution assays using vertebrate proteins, and genomic approaches on single cells should largely contribute to breakthrough findings in the field.

## Abbreviations

CDK, cyclin-dependent kinase; DDK, Dbf4-dependent kinase; ES, embryonic stem; G4, G-quadruplex; iPOND, isolation of proteins on nascent DNA; ORC, origin recognition complex; pre-RC, pre-replication complex; TAD, topological associated domain
